# Systematic Review and Meta-analysis of Prevalence of Coronary Artery Disease in Adult Patients with Cardiac Myxomas

**DOI:** 10.12688/f1000research.6641.1

**Published:** 2015-07-07

**Authors:** Matheus Silva, Matheus Carneiro, Júlio Nunes, Antônio da Silva, Marcos de Sousa

**Affiliations:** 1 Inteventional Cardiology Department, Hospital Universitário Ciências Médicas, Belo Horizonte, 30140-073, Brazil; 2 Inteventional Cardiology Department, Hospital São José do Avaí, Itaperuna, 28300-000, Brazil; 3 Post Graduation Program in Adult Health Sciences and Division of Cardiology and Cardiovascular Surgery, Hospital das Clínicas, School of Medicine, Universidade Federal de Minas Gerais, Belo Horizonte, Brazil

**Keywords:** Systematic review, meta-analysis, coronary artery disease, coronary angiography, myxoma

## Abstract

**Background**: Studies have reported varied prevalence estimates of coronary artery disease (CAD) in cardiac myxoma patients. We performed a systematic review and meta-analysis of observational studies to summarize the point prevalence of CAD in adults with cardiac myxomas.

**Methods and Results**: Two independent investigators searched MEDLINE and LILACS databases using the terms " *Myxoma*”, " *Coronary Angiography*" and " *Coronary Disease*" from inception through December 2014 for all relevant studies. We included 6 observational studies. Publication bias was evaluated through Egger's test and Trim and Fill method. A pooled estimate of CAD prevalence with corresponding 95% confidence interval (CI) was calculated based on a random-effects model. The pooled CAD prevalence in adult cardiac myxoma patients was 20.7% with low heterogeneity (I ^2^ = 14.86%).

**Conclusions**: It is a matter of debate if preoperative coronary angiography must be done as a routine procedure. Although coronary disease and angiographically detectable neovascularity can alter surgical management, more studies are needed to evaluate this question.

## Introduction

Myxomas are the most common primary cardiac tumors, although extremely rare. As an example, in one series of over 12,000 autopsies, only two were identified, for an incidence of less than 0.02 percent ^1^. Histologically, these tumors are composed of scattered cells within a mucopolysaccharide stroma. The cells originate from a multipotent mesenchyme that is capable of endothelial and neural differentiation ^2^. Myxomas produce vascular endothelial growth factor, which probably induces angiogenesis for tumor growth ^3^.

Macroscopically, the tumor surface can be smooth, friable or villous. The tumor diameter varies, ranging from 1 to 15 cm, with a weight typically between 15 and 180 g (mean, 37 g). Friable tumors are more prone to embolization, while larger tumors present with cardiovascular symptoms ^4^.

The mean age of patients with myxomas is 56 years and 64–70% are females. However, myxomas have been described in patients ranging in age from 3 to 84 years. Approximately 86% of all myxomas occur in the left atrium, and most of the remainder is found in the right atrium. Over 90% are solitary ^4, 5^.

The cardiovascular manifestations depend upon the anatomic location of the tumor. In a series of 112 consecutive cases of left atrial myxoma: (1) cardiovascular symptoms were present in 67%, more commonly in the form of mitral valve obstruction (mostly cardiac failure or malaise). Cardiac auscultation abnormalities occurred in 64%, essentially pseudo-mitral valve disease in 53.5% and more rarely the suggestive tumor plop in 15%. The most frequent electrocardiographic sign was left atrial hypertrophy in 35%, whereas arrhythmias were uncommon. (2) Embolic symptoms were observed in 29%, essentially cerebral emboli with stroke, with men at greater risk. (3) Constitutional symptoms were observed in 34% with fever, weight loss, or symptoms resembling connective tissue disease ^4^. Right atrial tumors are more commonly associated with signs and symptoms of right heart failure. Tumor fragments can embolize to pulmonary vasculature and cause symptoms consistent with pulmonary emboli, or in the presence of a patent foramen ovale or atrial septal defect, hypoxemia or systemic emboli ^6, 7^.

Echocardiography is a widely available, simple and noninvasive approach, which in almost all cases precisely locates the tumor and defines its extent. In addition, transesophageal echocardiography (TEE), cardiac magnetic resonance (MRI) and ultrafast computed tomography (CT) have also proved their usefulness in diagnosis ^8, 9^.

Once a presumptive diagnosis of a cardiac myxoma is made, surgical removal is indicated because of the risk of embolization or of sudden cardiac death. The prognosis for patients with solitary myxomas after surgical resection has been excellent with mortality rates of about 4%. Late recurrences are infrequent and reported to occur in 0.4–5% of patients ^10^.

Several studies have attempted to estimate the rate of cardiac myxomas with concomitant CAD and we therefore conducted a systematic review and meta-analysis of observational studies to summarize the point prevalence of CAD in adults with these tumors.

## Methods

We carried out a systematic review and meta-analysis of prospective and retrospective observational studies following the PRISMA statement (Supplementary Material S1) ^11^. Initially, a search in the main databases (MEDLINE, The Cochrane Library, and LILACS) was performed, searching for studies with similar objectives and methodology. No similar study was found.

A systematic MEDLINE search was performed with the medical subject headings (MeSH) terms (“Myxoma”[MeSH] AND “Coronary Angiography”[MeSH]) OR (“Myxoma”[MeSH] AND “Coronary Disease”[MeSH]), looking for trials in English, Spanish and Portuguese, published until December 2014, that performed coronary angiography in patients with cardiac myxomas. At the same time, a systematic LILACS search was also performed using the same MeSH terms and search strategy.

We designed a relatively strict set of inclusion and exclusion criteria and considered studies meeting these criteria to be of acceptable quality. The study selection criteria were: (1) observational studies, with prospective or retrospective data collection; (2) studies that provided a measure of CAD prevalence in adult patients with cardiac myxomas; (3) studies that included at least five cases of cardiac myxomas; (4) studies in which at least 75% of the adult cardiac myxoma patients had coronary angiographies; (5) angiographic and demographic data systematically reported.

Two researchers, according to the previously established inclusion criteria, then independently reviewed the titles returned by the systematic search. Exclusion by duplicity, title, abstract and full text analyses was independently performed and discrepancies in each stage were solved by consensus after discussion. The selected articles were read in full to confirm eligibility and their data was tabulated and reviewed for the statistical analysis. The second researcher independently double-checked the extraction of primary data from every study.

The meta-analysis of the pooled prevalence data, as well as associated graphic results was performed using the Comprehensive Meta Analysis software, version 2.2.064. Other computations were performed with IBM SPSS Statistics for Macintosh, Version 22.0.

Heterogeneity of accuracy measures was explored with the I ^2^ estimate (inconsistency measure) from Cochran *Q* according to the formula: I ^2^ = 100% x (Cochran *Q* – degrees of freedom)/Cochran *Q*. This describes the percentage of the variability in effect that is due to heterogeneity rather than sampling error (chance) ^12^. Publication bias was graphically assessed using funnel plot, Egger's test and Trim and Fill method ^13– 15^.

## Results

**Figure 1.**
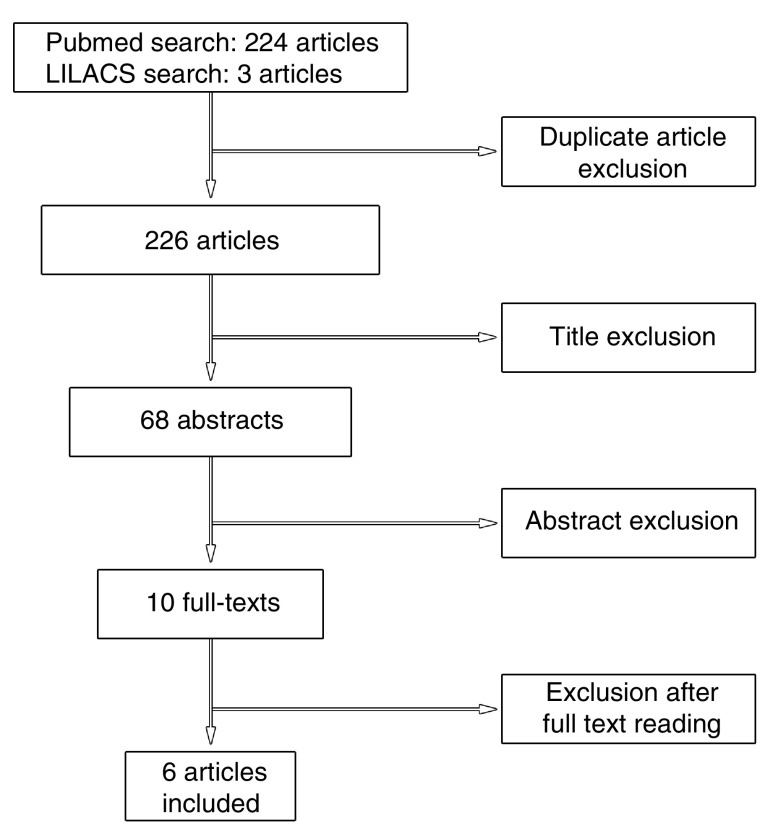
Flowchart of article exclusions by peer review.

A flow chart of the studies evaluation is shown in Figure 1. These latter studies were excluded because of a lack of angiographic data in at least 75% of patients with cardiac myxomas in the populations examined. Thus, a total of 6 studies evaluating the prevalence of CAD were selected according to the aforementioned criteria.

**Figure 2.**
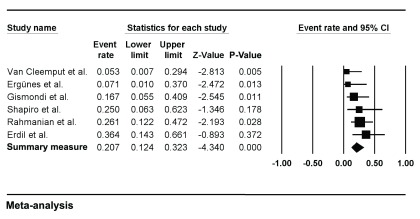
Forest plot of prevalence rate of CAD in adult myxoma patients in 6 studies. Horizontal lines represent 95% confidence intervals (CIs). Each box represents the prevalence rate point estimate, and its area is proportional to the weight of the study determined by inverse variance weighting. The *diamond* represents the overall summary estimate, with the 95% CI given by its width.

For each study, demographic characteristics, the proportion of adult cardiac myxoma patients who underwent coronary angiography and the location of the tumors are listed in Table I. The criteria used to define the presence of CAD and associated treatment when described is listed in Table II. The prevalence rates of clinically confirmed CAD for each of the 6 studies are reported in Figure 2.

**Table I.** Demographic characteristics of study populations, proportion of patients who underwent CA and location of tumors.

Study	Male/Female	Mean Age (Years ± SD)	Coronary Angiography	Intracardiac Location of Myxomas
Van Cleemput *et al.* ^16^	7/18	57.37 ± 9.97 (range 38–71)	19/25 (76%)	LA: 23 RA: 2
Ergünes *et al.* ^17^	8/9	54.64 ± 13.02 (range 27–75)	14/17 (82%)	LA: 14 RA: 3
Gismondi *et al.* ^18^	NP	NP	18/21 (86%)	LA: 17 RA: 1
Shapiro *et al.* ^19^	3/4	56.71 (range 52–65)	7/7 (100%)	LA: 7
Erdil *et al.* ^5^	4/7	55.72 (range 30–73)	11/11 (100%)	LA: 11
Rahmanian *et al.* ^20^	10/18	61.3 ± 13.5	23/28 (82%)	LA: 24 RA: 4

**Abbreviations:** CA, coronary angiography, LA, left atrium; RA, right atrium; NP, not provided.

**Table II.** Description of CAD patients in each individual study and associated treatment when presented.

Study	Description of CAD and Treatment
Van Cleemput *et al.* ^16^	One patient had significant CAD of the LCx, and was considered operable.
Ergünes *et al.* ^17^	One patient was determined to have CAD and was treated with additional CABG.
Gismondi *et al.* ^18^	Three patients had significant CAD, defined as the existence of a ≥ 50% diameter narrowing of the LMCA or a ≥ 70% diameter narrowing of the other coronary arteries.
Shapiro *et al.* ^19^	Two patients had significant coronary obstructions. One patient had a total obstruction of the RCA and an antecedent myocardial infarction. The second had a severe lesion on the LAD.
Erdil *et al.* ^5^	Four patients had concomitant CAD identified. At surgery CABG was performed after the resection of left atrial myxoma in three patients. The fourth patient had a noncritical lesion in the RCA and was treated medically.
Rahmanian *et al.* ^20^	Six patients had significant CAD, leading to percutaneous angioplasty and stent placement in three patients, and surgical revascularization during mass excision in the remaining three patients.

**Abbreviations:** CAD, coronary artery disease; LCx, left circumflex coronary artery; CABG, coronary artery bypass graft surgery; LMCA, left main coronary artery; RCA, right coronary artery; LAD, left anterior descending coronary artery; LA, left atrium; RA, right atrium; NP, not provided.

**Figure 3.**
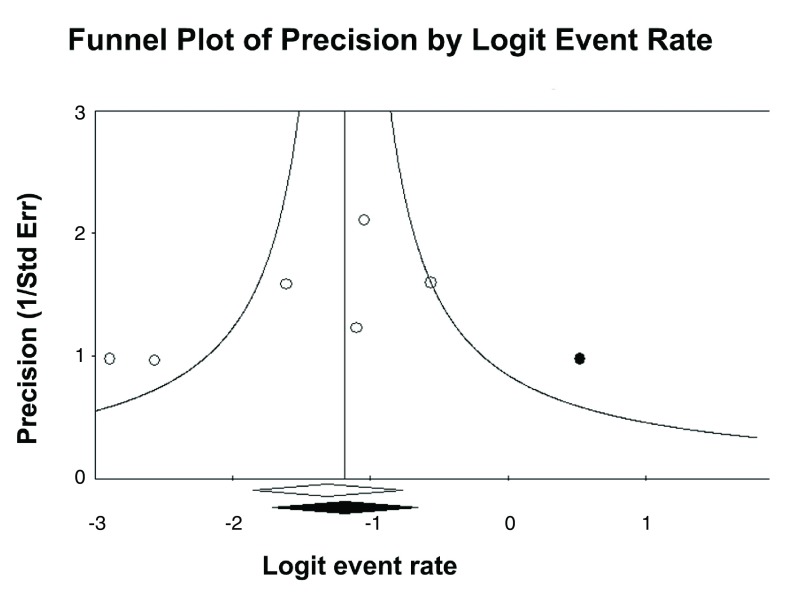
Funnel plot of precision by Logit Event Rate. This is a display of the study’s effect size on a logit scale against its precision for each study included in the meta-analysis. Egger's test P = 0.047. Trim and Fill method showed close observed (20.7% with 95% CI 12.4 to 32.3%) and adjusted (22.7% with 95%CI 13.0 to 36.5%) values.

As shown, we found an aggregated estimate of 20.7% (95% CI 0.12 to 0.32). The prevalence rates reported across these studies varied from 5.26% ^16^ to 36.26% ^5^ ( Figure 2), with low heterogeneity (Q-value = 5.873, P-value = 0.319, I ^2^ = 14.86%). Egger's test (two tailed) was borderline positive for publication bias (P = 0.047). A funnel plot with Trim and Fill method is shown in Figure 3. The close observed and adjusted values of pooled prevalence suggest a small influence of publication bias.

## Discussion

There is little documented literature on the relationship between CAD and cardiac myxomas ^8^. This article provides the first compilation of available angiographic data on adult patients with cardiac myxomas and CAD. According to this meta-analysis, the estimated prevalence of CAD in adult patients with myxomas is 20.7%, with low heterogeneity. In observational studies, Van Cleemput *et al.* ^16^, Ergünes *et al.* ^17^ and Gismondi *et al.* ^18^ found that CAD prevalence accounted for 5.3%, 7.1% and 16.6% of adult patients with myxoma, respectively. Shapiro *et al.* ^19^ found a CAD prevalence of 25% and Erdil *et al.* ^5^ of 36.4% of adult patients with myxomas. However, in these studies, the number of index patients considered was small, 7 and 11, respectively. Rahmanian *et al.* ^20^ evaluated 23 out of 28 adult myxoma patients with coronary angiography and found a CAD prevalence of 26.1%; nevertheless, this study enrolled the eldest patients of our series of studies (mean age, 61.3 years).

Concomitant coronary artery bypass grafting (CABG) surgery with resection of the tumor can be crucial in patients with critical coronary lesions. Erdil *et al.* ^5^ identified 4 patients out of 11 with concomitant CAD, 3 of which had adjuvant CABG performed, and a fourth, which had a noncritical lesion in the right coronary artery and was treated medically. Rahmanian *et al.* ^20^ also reported that in 6 out of 23 patients significant CAD was found leading to percutaneous angioplasty and stent placement in 3 patients, and surgical revascularization during mass excision in the remaining three patients. Indeed, out of the 14 patients with CAD identified in the 6 studies included in this meta-analysis, 12 (86%) were subject to, or were liable to invasive treatment.

The use of preoperative coronary angiography (CA) in adult myxoma patients is a topic of debate. Some argue that CA should only be performed in selected patients, particularly those aged > 35–40 years, with atherosclerotic risk factors, a positive anginal history or with a previous history of myocardial infarction to rule out concomitant coronary artery disease ^16, 20– 23^. However, many report that there has been no significant difference in symptoms, age or prevalence of coronary risk factor distribution between myxoma patients who present with CAD and those who do not ^5, 18, 24, 25^. Actually, even patients without any risk factors can present with CAD ^25^. Therefore, others suggest that all adult patients diagnosed with myxomas should undergo CA ^5, 8, 18, 25, 26^. In fact, preoperative CA seems to be quite safe; thus far there has been no report of procedure-related complications ^8, 16, 18, 24– 27^.

Preoperative CA can yield even more information that may prove useful intraoperatively ^8^. Selective CA occasionally may visualize the tumor by revealing the angiographic sign of ‘tumor vascularity’, first described by Marshall *et al.* ^28^, which consists of clusters of small and tortuous vessels with blood pooling and tumor blush arising from the coronary arteries supplying the tumor ^26, 27^. From the experience of Van Cleemput *et al.* ^16^ and the data published by Fueredi *et al.* ^27^ and Chow *et al.* ^26^, angiographically visible neovascularity is prevalent in around 40% of symptomatic cardiac myxoma patients. This finding suggests a tumoral origin of the mass, however not specific. Systematic performance of preoperative CA has been recommended by some authors in an attempt to identify a large supplying vessel ^20^.

Failure to identify and ligate these vessels may lead to a coronary-cavitary fistula ^29^ or a “steal syndrome”, by re-directing blood from a coronary artery into a cardiac chamber, with consequent myocardial ischemia ^8^.

Our study has limitations. As not all patients in each study had performed coronary angiography, the pooled prevalence can be overestimated by verification bias. Patients not selected to perform CA are probably those with a low risk profile. To overcome this limitation, we decided to not include those studies that reported less than 75% of patients submitted to CA. Since it is a rare condition, samples are small. Also, number of studies is small. On the other hand, a systematic review is one way to gather evidence on rare conditions. Although the publication bias test was borderline positive, the Trim and Fill method suggested small or no influence of publication bias on pooled results. Even if it is still present, prevalence could be higher, since the adjusted value is slightly higher than the observed value.

## Conclusion

Routine preoperative angiography in all cases of these tumors is still a matter of debate. Pooled prevalence of coronary disease and the potential to disclose angiographically detectable neovascularity are arguments to advocate routine angiography. Patient management and clinical outcomes could be potentially altered, but more studies are needed to answer this question.
