# Supplementary material for: Systematic Review and Meta-analysis of Prevalence of Coronary Artery Disease in Adult Patients with Cardiac Myxomas
Source: F1000Res. 2015 Jul 7;4:194. [Version 1] doi: 10.12688/f1000research.6641.1 (PMC5461895; doi:10.12688/f1000research.6641.1)
Supplement: Peer review history

# Peer review: Referee response for version 1

**DOI:** 10.5256/f1000research.7135.r22954

**Published:** 2017-05-31

**Authors:** Christian Röver

**Affiliations:** 1 Department of Medical Statistics, University Medical Center Göttingen (UMG), Göttingen, Germany

The meta-analysis seems to be performed properly, I only have a few remarks concerning mostly the presentation of results.

 Methods section:

 1) It would suggest mentioning explicitly here that:

    a) a random-effects model was used.

    b) the DerSimonian-Laird heterogeneity estimator was used.

    c) a logit- (or log-odds-) transformation was used for the effects.

 Results section:

 2) The forest plot (Fig.2) is somewhat confusing:

    a) the x-axis should not extend beyond 0 and 1.

    b) the p-values (and z-values) shown apparently relate to a test of zero log-odds (i.e. a prevalence of 50%), which does not seem to make sense here; these should be dropped.

    c) it would be good to also show the numbers of events and totals (CAs and CADs) either here or in one of the tables.

 3) I do not see why a selection effect would be plausible, and why one would perform Eggers' test here. Would it be plausible that certain results would not be accessible because they would exhibit either a very high or very low CAD prevalence? If not, then the test (and the funnel plot) should probably be omitted.

 General minor comments:

 4) In order to put the results into context, I would suggest to also hint on the order of magnitude of CAD prevalence in non-myxoma populations, or on potential CAD prevalence thresholds that might warrant consequences.

 5) The studies in tables I and II (last two rows) are in a different order than in Fig.2

I have read this submission. I believe that I have an appropriate level of expertise to confirm that it is of an acceptable scientific standard.

# Peer review: Referee response for version 1

**DOI:** 10.5256/f1000research.7135.r9393

**Published:** 2015-08-24

**Authors:** Guilherme Costa

**Affiliations:** 1 Cancer Research UK Stem Cell Biology Group, Cancer Research UK Manchester Institute, University of Manchester, Manchester, UK

Definitely an important and clinically relevant topic. Through accurately performed meta-analysis and systematic review, the authors remind us of a rare and usually underrated - though certainly morbid - condition which could be managed differently if better researched on.

I have read this submission. I believe that I have an appropriate level of expertise to confirm that it is of an acceptable scientific standard.
